# Storage fidelity for sequence memory in the hippocampal circuit

**DOI:** 10.1371/journal.pone.0204685

**Published:** 2018-10-04

**Authors:** Mehdi Bayati, Torsten Neher, Jan Melchior, Kamran Diba, Laurenz Wiskott, Sen Cheng

**Affiliations:** 1 Institut für Neuroinformatik, Ruhr-University Bochum, Bochum, Germany; 2 Mental Health Research and Treatment Center, Department of Clinical Child and Adolescent Psychology, Faculty of Psychology, Ruhr University Bochum, Bochum, Germany; 3 Department of Anesthesiology, University of Michigan, Ann Arbor, United States of America; Plymouth University, UNITED KINGDOM

## Abstract

Episodic memories have been suggested to be represented by neuronal sequences, which are stored and retrieved from the hippocampal circuit. A special difficulty is that realistic neuronal sequences are strongly correlated with each other since computational memory models generally perform poorly when correlated patterns are stored. Here, we study in a computational model under which conditions the hippocampal circuit can perform this function robustly. During memory encoding, CA3 sequences in our model are driven by intrinsic dynamics, entorhinal inputs, or a combination of both. These CA3 sequences are hetero-associated with the input sequences, so that the network can retrieve entire sequences based on a single cue pattern. We find that overall memory performance depends on two factors: the robustness of sequence retrieval from CA3 and the circuit’s ability to perform pattern completion through the feedforward connectivity, including CA3, CA1 and EC. The two factors, in turn, depend on the relative contribution of the external inputs and recurrent drive on CA3 activity. In conclusion, memory performance in our network model critically depends on the network architecture and dynamics in CA3.

## Introduction

The hippocampus has been implicated in the acquisition and consolidation of memories in a variety of paradigms, for instance: episodic memories in humans [1, 2], associating time-delayed stimuli in rats [3], paired-associate memory even in the absence of a delay [4], and spatial memory [5]. However, it remains unclear, how the hippocampal circuit stores and retrieves memories. Based on its anatomical and physiological properties, the hippocampus can be divided into the DG, which includes a large number of small granule cells with low activity [6], and the CA3, CA2 and CA1 regions consisting of a homogeneous set of pyramidal cells. The connections between the subregions are established in a feedforward manner [7]. CA3 is well-known for its recurrent collaterals [8, 9], which play a key role in memory retrieval. The CA3 region has been suggested to function as an auto-associative memory, performing pattern completion when a partial and/or noisy cue is provided [10–15]. The attractors in the recurrent CA3 network are thought to be established rapidly when cortical inputs drive activity and plasticity in CA3. Over the last decades, this model has become known as the standard framework [16] and it continues to drive hippocampal research forward.

However, the experimental support for the standard framework remains mixed. On the one hand, it is bolstered by observations that rats with lesioned CA3 are impaired in remembering a location when parts of the spatial cues are removed [17] and that spatial pattern completion apparently requires plasticity in the recurrent CA3 synapses [18]. On the other hand, the standard framework cannot readily account for observations of numerous types of sequential neural activity in the hippocampal formation, because CA3 dynamics is designed to reach stable attractor states [19]. For instance, multiple studies implicate the hippocampus in temporal sequence learning. Rats with hippocampal lesions have difficulty remembering sequences of spatial locations [20] and hippocampal lesions impair a rat’s ability to learn which odor came first in a sequence of odors [21]. However, they were unimpaired at recognizing whether a particular odor had previously appeared in the experiment, or not. Similarly, animals with CA1 lesions have difficulty in disambiguating the temporal order of stimuli, particularly when they happened close together in time [22]. Agster et al. [23] showed that hippocampal rats had deficits disambiguating overlapping odor sequences. More generally, subsequent studies have shown that the medial temporal lobe (MTL) is involved in associating discrete items and their contexts across time and/or space [24, 25]. Electrophysiological studies have revealed further evidence that temporal sequences might be intimately tied to the hippocampus. After rats run through the place fields of hippocampal CA1 place cells causing the place cells to fire in a certain order, the cells become active in the same sequences during immobility awake states or sleep [26, 27]. This phenomenon has been called replay [28–31].

The generation of neuronal sequences in recurrent neural networks has been extensively studied in general computational models [32–37] and in models of the hippocampus [38, 39]. Levy and colleagues used sparsely connected random networks as a model of CA3 [38]. This model provides a unified computational framework that accounts for a number of hippocampal sequence processing tasks, e.g., sequence completion with an ambiguous subsequence, jump-ahead recall, finding a short cut, etc. It has also been suggested that neural codes in the hippocampus are organized by theta and gamma oscillations [39–41]. The aforementioned CA3 models focus on rather artificial memory patterns, which are either random or correlated in a systematic fashion. By contrast, neural activity patterns in the input region of the hippocampus, the entorhinal cortex (EC), have a unique non-random structure, for instance from grid cells [42], with very different correlations than standard noise distributions [43]. Furthermore, the models focus on an isolated CA3 network, thus neglecting the inevitable encoding and decoding in feedforward projections. It has been shown computationally that associative projections are capable of reconstructing the memory of grid cell patterns even when the recurrent connections (auto-associative function) in CA3 are removed [43]. This study illustrates how essential it is to consider the whole hippocampal loop while investigating individual functional roles of the subregions.

We have recently suggested an alternative theory of how neural sequences might be stored in the hippocampus [19], called CRISP (Content Representation, Intrinsic Sequences, and Pattern completion). In this framework, neural sequences are intrinsically generated in CA3. To store episodic memories, sequences of external input patterns are mapped onto these intrinsic CA3 sequences through synaptic plasticity in the feedforward projections (e.g., [44]). Here we develop a computational model of the cortico-hippocampal circuit (consisting of the EC-CA3-CA1-EC loop) to study the storage and retrieval of sequence memory. The neural network architecture is largely derived from our previous work [43], which in turn was adopted from Fontanari et al. (1995) [45] with the important exception of the CA3 recurrent dynamics. Nevertheless, storing sequences presents entirely different challenges from storing static patterns. We focused on two aspects that are key in the CRISP theory. First, what is the computational advantage, if any, of generating sequences intrinsically in CA3? We previously argued that limited plasticity in CA3 during memory encoding is a better match to experimental findings [19, 46], but this hypothesis has not been studied computationally before. Here, we test recurrent CA3 networks that are driven to a different degree by intrinsic dynamics vs. external inputs for their ability to robustly generate sequences of activity patterns. Second, how do correlations due to CA3 dynamics affect pattern completion in the complete circuit? Unlike the CA3 recurrent network, the feedforward connectivity between the hippocampal subregions [7] has received much less attention until recently [43, 47]. We previously found that the degree of spatial correlations in the EC inputs determine whether a recurrent or a feedforward network architecture is better at performing pattern completion [43].

We find that two factors have a strong influence on overall memory performance in our model: the robust retrieval of sequences from CA3 and the network’s ability to perform pattern completion through the feedforward connectivity in the hippocampal circuit. Both of these factors, in turn, depend on the relative influence of EC feedforward and recurrent inputs on CA3 activity. So, the cortico-hippocampal circuit can robustly store and retrieve sequences of patterns, but memory performance critically depends on the network architecture and dynamics in CA3.

## Materials and methods

**Fig 1:**
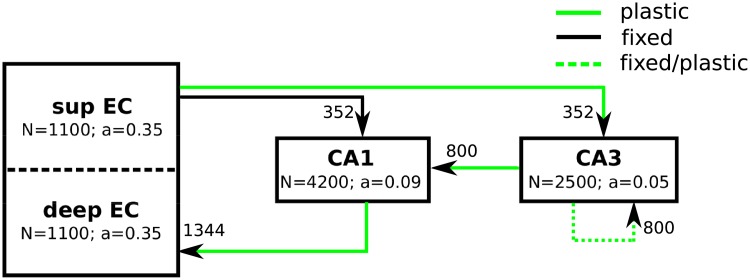
Schematic of the model. The three subregions EC, CA3 and CA1 are included in the model. The parameter *a* denotes the proportion of cells that are active, on average, at any given time. Arrows indicate connectivity between regions. Solid black lines indicate fixed random connections, solid green lines represent plastic connections that are adjusted during learning, and dashed lines show connections that could be either fixed (using hand-wired models for CA3) or plastic (using EC-input and intrinsic input to train CA3 weights). The number next to the arrows shows the number of connections that one cell receives from cells in the upstream region.

In this study, we store and retrieve sequences of external input patterns in a neural network, which represents the hippocampal formation. The model includes the entorhinal cortex (EC), CA3 and CA1 (Fig 1).

### Input statistics

To test the ability of each network model to store memory sequences, we generate *P* = *L* × *M* patterns, where *L* is the number of sequences, each with *M* patterns. We denote the set of input patterns as

$$\begin{array}{c} \{u_{l,m}:1\leq l\leq L,1\leq m\leq M\}. \end{array}$$ (1)

**Fig 2:**
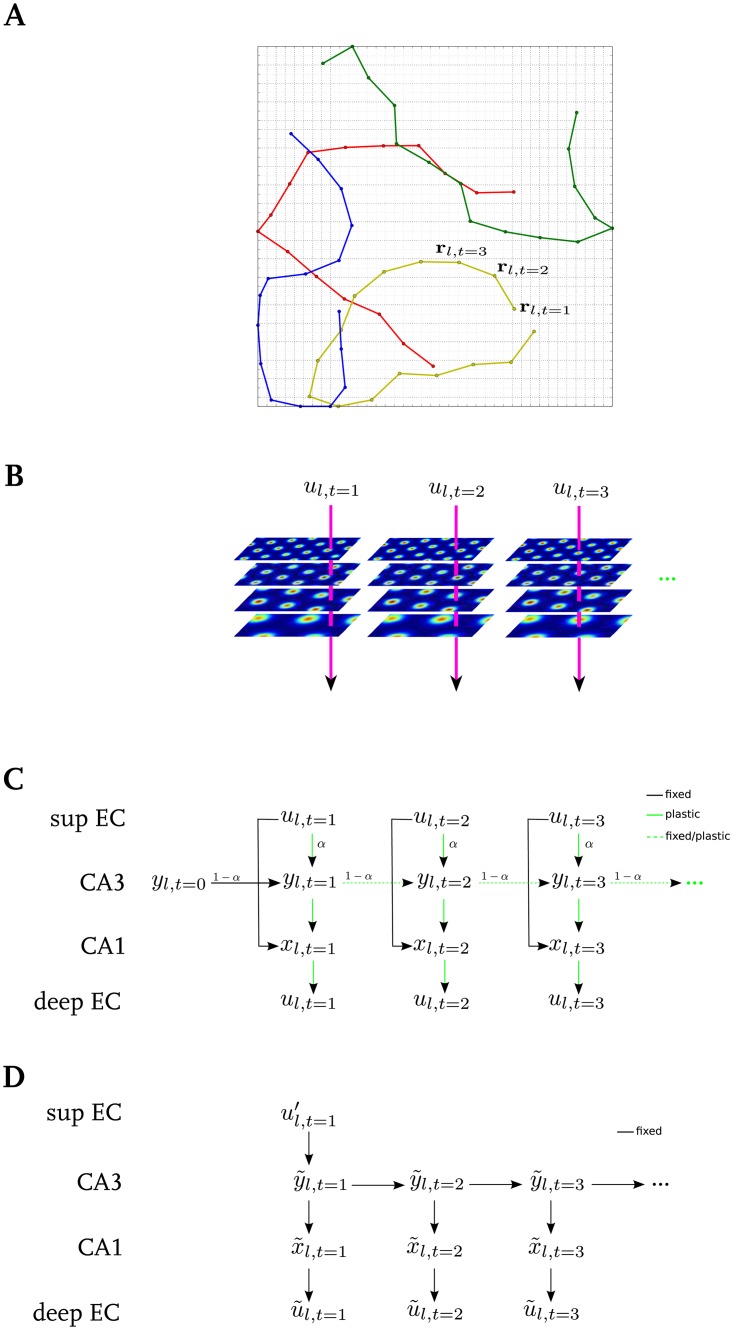
Schematic of memory storage and retrieval. **A**: Four example trajectories that are traversed in the environment. **B**: Illustration of the firing rate map of four example grid cells (one from each module) and three population patterns *u*_*l*,*t*_ (arrows) at example locations **r**_*l*,*t*_. **C**: To store a sequence (*u*_*l*,1_, …, *u*_*l*,*M*_) that represents an episodic memory, a sequence (*y*_*l*,0_, *y*_*l*,1_, …, *y*_*l*,*M*_) is activated in CA3 and each element *u*_*l*,*t*_ is associated with a particular CA3 state *y*_*l*,*t*_. The CA3 dynamics is initialised externally with an initial pattern (*y*_*l*,*t* = 0_), which is a random pattern for the DDN and RCN and a local bump-shape pattern in a random location for the LCN. When the DDN model is used, the successive states of CA3 at *t* ≥ 1 are associated together, otherwise CA3 connections remain fixed. The solid green and black lines between the areas indicate the associations between patterns and fixed random connections, respectively. Furthermore, the feedforward connections EC-CA3, CA3-CA1, and CA1-EC are adjusted to associate the activity patterns between the respective regions. **D**: Retrieval of a stored memory sequence from CA3 based on a partial input cue $u_{l,t=1}^{{}^{\prime }\text{EC}}$. Note that in the retrieval phase all connections are fixed. The first pattern in CA3 is driven directly from EC and CA3 dynamics retrieves the rest of the sequence. The retrieved elements (patterns) are noisy and are cleaned up by the CA1–EC network.

Since we recently found that the statistics of the stored patterns has a large impact on the memory performance of a network [43], we consider more realistic inputs in EC. As the animal traverses the environment (Fig 2A), the activity of a grid cell in the medial entorhinal cortex (MEC) of many species is modulated by the location of the animal such that discrete firing fields are arranged in a periodic hexagonal grid (Fig 2B) [42]. At each spatial location **r**_*l*,*t*_, the population of grid cells form an activity pattern *u*_*l*,*t*_ (Fig 2B), the sequence of which are stored in our hippocampal model. According to experimental findings the grid cell population is divided into four modules [48]. Cells in the same module have similar grid spacing and orientation, but different spatial phases, which were drawn from normal distributions. The mean grid spacings *s*_*i*_ and orientations of the modules are 38.8, 48.4, 65, 98.4 cm, and 15, 30, 45, 60 degrees. For each grid cell, these parameters are drawn from normal distributions with standard deviations of 8 cm and 3 degrees, respectively. See [43], Fig 1B and 1C for the resulting distribution of spacings and orientations of the population. Most grid cells (87%) belong to the two modules with small spacings [48].

The activation of grid cell *i* at location **r** = (*x*, *y*) is determined by

$$\begin{array}{c} h^{i}\left(r\right)=A^{ij}\exp[-\ln\left(5\right){\left(\frac{d(r)}{\sigma^{i}}\right)}^{2}], \end{array}$$ (2)

where *d* is the Euclidean distance to the nearest field center *j* and *σ*^*i*^ is the radius of the firing field. Each field has the same size, which is related to the grid spacing via *σ*^*i*^ = 0.32*s*^*i*^ (see Fig S4G in [42]). *A*^*ij*^ is the peak firing rate of the cell in the center of a field and reaches 0.2*A*^*ij*^ at the border, which is motivated by the definition of a place field [42]. The peak firing rates *A*^*ij*^ are drawn from a normal distribution with mean 1 and standard deviation 0.1 (see [43] for a visualization of grid patterns). At any location, a binary activity pattern is generated by setting the *k* cells with the highest activation to one and all others to zero according to Eqs 6 and 7.

To generate a sequence of input patterns, we simulate the spatial behavior of a rodent running in a 1m × 1m square environment. The trajectory is generated as follows. The agent is placed at a randomly chosen starting point and then traverses the environment with a constant speed of *v* = 10 cm per time step. In each time-step, the position of the agent **r**_*t*_ is determined by

$$\begin{array}{ccc} r_{t} & = & r_{t-1}+v\frac{m_{t}}{∥m_{t}∥} \end{array}$$ (3)

$$\begin{array}{ccc} m_{t} & = & \left(1-\mu \right)m_{t-1}+\mu \varepsilon_{t}, \end{array}$$ (4)

where **m**_*t*_ is a velocity vector, momentum *μ* modulates the smoothness of the path, which is set to *μ* = 0.4, and *ε*_*t*_ is a random vector with two elements each drawn from a uniform distribution between −1 and 1. Whenever the virtual animal hits the boundaries, in the next time-step it turns into the environment with a random direction. Finally, the trajectory positions are discretized by replacing them with the closest node on a 40 x 40 lattice laid over the environment. Using the model described above we generate sequences of input patterns *u*_*l*,*m*_, which represent the activity of grid cells when an animal explores a novel environment.

The dynamics of neural networks in the brain is continuous and the time constants governing synaptic conductances are in the range of tens of milliseconds. Here, we study sequence memory encoding and retrieval at this fast time scale. To relate these neural sequences to behavioral sequences on the order of seconds or more, we assume that there is sequence compression mechanism outside the storage network, such as, e.g., theta phase precession [49]. Theta phase precession is already present in the input to the hippocampus, in the medial entorhinal cortex [50], suggesting that behavioral sequences arrive in the hippocampus temporally compressed. The sequence compression mechanism is outside the scope of this paper, since we focus on the storage and retrieval of neural sequences.

### Model architecture and activation function

The number of neurons *N* in each region (Fig 1) is based on anatomical data from the rat hippocampal formation [7, 51] and is scaled down by the factor of 100 in order to reduce the computational costs (see [43] for details). Neurons in our model are binary, i.e., they are either active or silent reflected by a value of 1 or 0, respectively [45]. The activation *h*^*i*^ of the receiving cell *i* is calculated as the weighted sum of its inputs *u*^*j*^

$$\begin{array}{c} h^{i}=\sum_{j=1}^{N_{\text{in}}}w^{ij}u^{j}, \end{array}$$ (5)

where *w*^*ij*^ is the strength of the connection from cell *j* to cell *i* and is set to 0 when a connection does not exist. Inhibitory cells are not modeled explicitly but rather through their effect on a population level [52–56]. A *k*-Winner-Take-All (kWTA) mechanism is applied to determine which cells become active. The *k* cells with the highest activation are set to 1 and the others are inhibited and thus set to 0, i.e.,

$$\begin{array}{ccc} \kappa & : & R_{+}^{N}\times N\to {\left\{0,1\right\}}^{N} \end{array}$$ (6)

$$\kappa^{i}\left(h;k\right)=\left\{ \begin{array}{cc} 1 & \;\text{if}\;h^{i}\;\text{is}\;\text{among}\;\text{the}\;k\;\text{highest}\;\{h^{j}:1\leq j\leq N\}.\\ 0 & \;\text{otherwise}. \end{array}\right.$$ (7)

The number *k* is chosen uniformly from the interval [(1 − *δ*)*aN*, (1 + *δ*)*aN*], ensuring that a varying number of *k* cells is recruited in different patterns. The parameter *a* is the sparsity in the corresponding region (Fig 1) and *δ* = 0.15.

The number of connections that a neuron receives from upstream neurons is scaled up by a factor $\sqrt{100}$ from the rat hippocampus (Fig 1). This ensures that a sufficient number of connections exist to store the sparse patterns in the network. In particular, the sparse connectivity in rat CA3 (< %3) [15] turns into 32% after scaling. We confirmed that our qualitative results do not depend sensitively on the connectivity parameter by running our simulations for more diluted and denser connectivities. The initial weights for the feedforward EC-CA3 and EC-CA1 projections are randomly sampled from a uniform distribution between zero and one. The weights of the CA3-CA1 and CA1-EC projections are initialized to zero since they will be learned in the learning phase. Initialization of the recurrent CA3 weights will be described below.

Recent findings indicate that reciprocal interactions between deep and superficial layers of the EC are quite substantial [57, 58]. Furthermore, main cortical inputs target both deep and superficial layers. Thus, the deep and superficial layers of the EC might act as a single functional entity, rather than as separate structures [59]. Therefore, the superficial and deep layers of the EC are clamped to the same activity during learning. Please note that this does not mean that we close the loop, namely the activities in the EC outputs do not propagate, via the EC input, through the network.

### Models of CA3

The dynamics of CA3 is crucial for the overall function of the model, since only CA3 stores and retrieves the sequential aspect of the memory sequences. To study the different roles of external inputs and recurrent connections in driving CA3 network states, we systematically vary the relative contributions of the two inputs (see the first model below). This model is the main focus of our current study. To explore some of our findings in more constraint settings, we contrast this main model with two further models, which produce CA3 sequences entirely intrinsically and do not exhibit plasticity in recurrent CA3 connections. The second model generates sequences of uncorrelated patterns, whereas the third model generates sequences of highly correlated patterns. In all networks, non-existing connections are modeled as connections with zero weight.

1. *Dual-driven network (DDN)*: Each CA3 node is connected randomly to 32% of the other nodes. The weights for the connections are sampled from a uniform distribution between zero and one. CA3 activity patterns are driven jointly by EC inputs and CA3 collateral inputs *during the learning phase*. We use the mixing parameter (0 ≤ *α* ≤ 1) to control the contribution of these inputs to the activation of a CA3 cell. The activation *h*^*i*^ of the receiving cell *i* depends on the activity of the CA3 network in the previous time step and the concurrent activity in EC.
  $$\begin{array}{c} h_{t}^{i}=\left(1-\alpha \right)\sum_{j=1}^{N_{\text{in}}^{\text{CA}3}}w^{ij}y_{t-1}^{j}+\alpha \sum_{k=1}^{N_{\text{in}}^{\text{EC}}}w^{ik}u_{t}^{k} \end{array}$$ (8)
  When *α* = 0 the network activity is driven intrinsically. On the other hand, when *α* = 1, the network activity is driven entirely by EC inputs. Intermediate values of *α* integrate contributions of both inputs. The CA3 recurrent network learns the sequences through successive hetero-associations, i.e., each network state is associated with the subsequent state of the CA3 network (see subsection *Learning Phase*). Note that here we study sequence memory storage in CA3, which is different from its suggested auto-associative function, where individual, single patterns are stored in the network.
2. *Randomly connected network (RCN)*: The connectivity is initialized randomly like in the DDN, but the weights in this model remain fixed during the learning phase. CA3 activity is driven intrinsically, i.e., described by Eq 8 with *α* = 0.
3. *Locally connected network (LCN)*: Each CA3 node is assigned a virtual location in a 2-d square environment and connected to 800 of its nearest neighbours. The weights of these connections are assigned according to a Gaussian kernel based on the distance between cells. Such a continuous attractor network generates a bump of activity. We introduce an adaptation parameter (0 ≤ *J* ≤ 1) to destabilize the bump of activity. The adaptation term forces the bump to move through the network [46]. The adaptation parameter controls the speed of the bump movement. In summary, the activation *h*^*i*^ of the receiving cell *i* depends on its activity in the previous time step and the weighted sum of its recurrent inputs *y*^*j*^.
  $$\begin{array}{c} h_{t}^{i}=\left(1-\alpha \right)\left(1-Jy_{t-1}^{i}\right)\sum_{j=1}^{N_{\text{in}}^{\text{CA}3}}w^{ij}y_{t-1}^{j}, \end{array}$$ (9)
  where we use the factor (1 − *α*) to be consistent with Fig 2C, but *α* = 0 in all simulations. Note that there are no periodic boundary conditions in the LCN. The neurons at the boundaries receive the same number of inputs as the neurons in the center of the sheet. This slightly slows down the diffusion of the activity bump near the boundaries of the LCN.

During the learning phase, when input patterns from EC are hetero-associated with network states in CA3, the initial CA3 pattern has to be triggered externally. The initialization pattern is adjusted according to the CA3 model: the DDN and RCN are initialised with a random pattern; and for the LCN, we use a local bump-shaped pattern in a random location. Once initialized, the next pattern in CA3 is generated according to Eqs 8 or 9, and 6 and 7 (also see Fig 2C).

### Learning phase

Our goal is to store a set of sequences {*u*_*l*,*m*_: 1 ≤ *l* ≤ *L*, 1 ≤ *m* ≤ *M*} in the network such that they can be retrieved as accurately as possible. To store a sequence of patterns during the learning phase, the plastic weights between subregions (green arrows in Fig 1) are adjusted according to Hebbian learning (Eq 10). For a hetero-association of a pre-synaptic pattern *a* with post-synaptic pattern *b*, we use the so-called Stent-Stinger rule [60]

$$\begin{array}{c} w^{ij}=c^{ij}\sum_{l=1}^{L}\sum_{m=1}^{M}\left(a_{l,m}^{j}-{\bar{a}}^{j}\right)b_{l,m}^{i}. \end{array}$$ (10)

*c* denotes the connection matrix between two regions, i.e., *c*^*ij*^ = 1 if there is a connection from cell *j* to *i* and *c*^*ij*^ = 0 otherwise. It insures that non-existing connections remain at zero weight. ${\bar{a}}^{j}$ is the mean activity level of the pre-synaptic cell over all sequences. To store sequences, we first apply the input patterns *u*_*l*,*m*_ to EC. The activities in CA3 are generated according to the model used and as described in subsection. The sequence of CA3 patterns **y** are then hetero-associated with the EC inputs **u** (Eq 10). Neural activity in CA1, **x**, is triggered by the constant EC input weights via Eqs 5–7 (Fig 2C). Furthermore, the patterns in CA3 are hetero-associated with the patterns in CA1, and the CA1 patterns with input patterns **u** in the EC output (Eq 10).

The weights in CA3 are plastic only in the DDN model. The CA3 patterns are first driven jointly by recurrent and external EC inputs (Eq 8). Then, the corresponding patterns between the CA3 and EC and successive patterns in the CA3 are hetero-associated based on Eqs 10 and 11, respectively. During the learning phase for the DDN, we adjust the recurrent weights *v*^*ij*^ in CA3 according to the co-variance rule [61] to learn hetero-associations among a set of patterns in a sequence {*y*_*l*,*m*_: 1 ≤ *l* ≤ *L*, 1 ≤ *m* ≤ *M*}.

$$\begin{array}{c} v^{ij}=c^{ij}\sum_{l=1}^{L}\sum_{m=1}^{M-1}\left(y_{l,m}^{j}-{\bar{y}}^{j}\right)\left(y_{l,m+1}^{i}-{\bar{y}}^{i}\right). \end{array}$$ (11)

By subtracting the mean, the two learning rules model LTP and LTD. Furthermore the subtraction is essential for computational reasons (for example, see chapter 8.2 of [62]). After applying the learning rules, the Euclidean norm of the vector *w*^*i*^ of incoming weights to cell *i*, in all layers, is normalized to one to ensure that not always the same cells are activated.

### Retrieval phase

After sequences have been stored in the network, we initiate recall by setting EC to a noisy recall cue $u_{l,1}^{\prime }$. This cue is generated by modifying the first pattern of the stored sequence *u*_*l*,1_. A number of active neurons are chosen randomly and inactivated. The same number of silent neurons is chosen randomly and set to be active. Therefore, the number of active neurons is preserved in all cue patterns. The quality of the recall cue is controlled by the number of cells that fire incorrectly. It is measured by the Pearson correlation between the original pattern and the recall cue (*C*_cue_) (see below). This model of retrieval corresponds to placing the animal at a previously visited location, but the grid cell population activity at that location might not perfectly match the pattern during the previous visit because of internal noise or slight changes in the external environment. We test the model performance with 6 different levels of average recall cue qualities, namely *C*_cue_ ∈ {0, 0.2, 0.4, 0.6, 0.8, 1}. The actual cue quality of a particular cue varies slightly depending on the number of cells, the sparsity of the pattern, and how many cells have the incorrect activity. Hence, negative recall cue qualities are possible as well. The recall cue triggers a pattern ${\tilde{y}}_{l,1}$ in CA3 directly via the previously learned weights from EC to CA3, which subsequently generates an intrinsic sequence (${\tilde{y}}_{l,2},{\tilde{y}}_{l,3},\ldots$). This sequence is transferred to CA1 (${\tilde{x}}_{l,1},{\tilde{x}}_{l,2},\ldots$) and from CA1 back to EC (${\tilde{u}}_{l,1},{\tilde{u}}_{l,2},\ldots$). Fig 2D illustrates the retrieval process in our model. The synaptic weights remain fixed during the retrieval process.

### Analysis

#### Retrieval quality

To measure how well a retrieved pattern matches the stored pattern in any region, we use the Pearson correlation between the originally stored pattern *a*_*l*,*m*_ of a sequence and the retrieved one ${\tilde{a}}_{l,m}$. It is defined as

$$\begin{array}{c} C\left(a_{l,m},{\tilde{a}}_{l,m}\right)=\frac{{\left(a_{l,m}-\bar{a}\right)}^{T}\left({\tilde{a}}_{l,m}-\bar{\tilde{a}}\right)}{∥a_{l,m}-\bar{a}∥\cdot ∥{\tilde{a}}_{l,m}-\bar{\tilde{a}}∥}, \end{array}$$

where $\bar{a}$ and $\bar{\tilde{a}}$ are the means of the stored and retrieved patterns over all sequences, *l* = 1, 2, …, *L* and *m* = 1, 2, …, *M*, respectively. The higher the value of this correlation is, the more similar the recalled pattern is to the original one. We refer to the retrieval quality in CA3, CA1 and the output in EC as *C*_CA3_, *C*_CA1_, and *C*_EC_, respectively.

#### Pattern completion

Pattern completion is defined as the retrieval of additional information from a memory network that was not present in the recall cue. To measure pattern completion in our model, we compare the retrieval quality at some stage $C(b_{l,m},{\tilde{b}}_{l,m})$ to the retrieval quality at the next stage $C(a_{l,m},{\tilde{a}}_{l,m})$. Here, the stages correspond either to two connected layers in a feedforward network, or to subsequent network states in the recurrent CA3 network.

**Fig 3:**
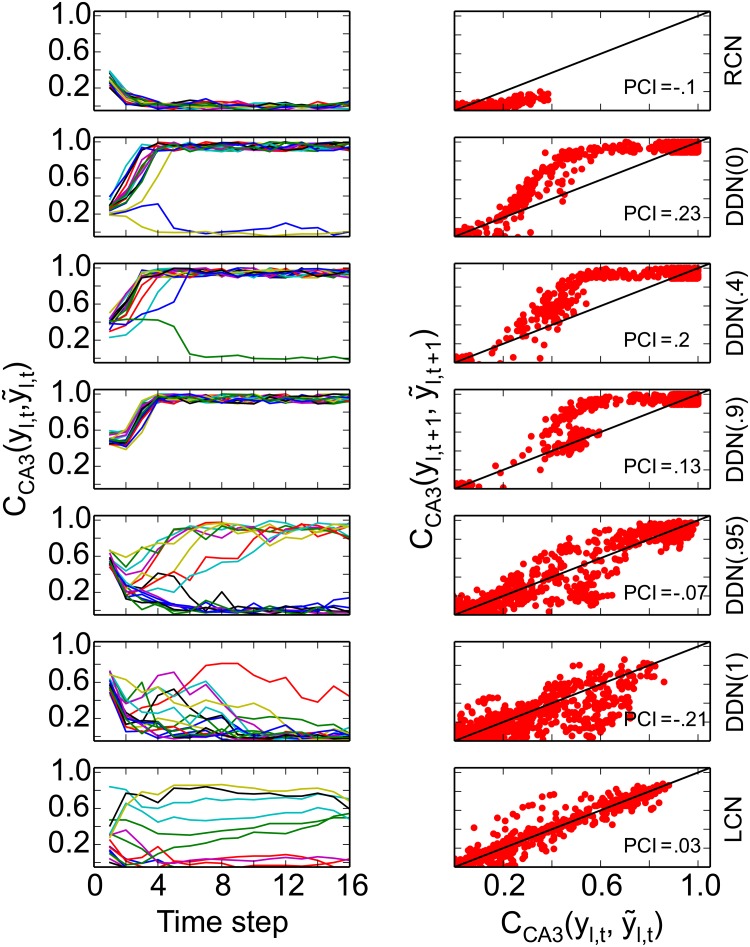
Recall performance in CA3. **Left**: Each panel on the left column shows an example of the retrieval quality in a CA3 model as labelled on the right hand side of the figure, when recall cue with cue quality *C*_cue_ = 0.4 is provided to EC. The horizontal axes represent the position of the pattern in the sequence. Each colored line shows the correlation between the retrieved and the correct patterns in a sequence. The RCN (first row) is highly sensitive to noise, whereas the LCN (last row) seems to keep the same input information while retrieving the stored sequence. DDN(*α* ≤ 0.9) shows the best performance and retrieves the previously stored sequence from the noisy input cue. **Right**: Summary of recall performance in CA3 for the entire range of noise levels. The pattern completion plot for different network models. Data-points above the diagonal indicate good sequence completion, whereas data below the diagonal indicate that information in the input is lost. Data on the diagonal show that the network maintains the information in the input cue along the sequence. Overall, DDN(*α* ≤ 0.9) performs best, even with highly corrupted cues.

To perform the comparison, we make a scatter plot of $C(b_{l,m},{\tilde{b}}_{l,m})$ vs $C(a_{l,m},{\tilde{a}}_{l,m})$ for all pairs of stored and retrieved patterns. If the points line up along the identity line, then the processing does not add any information and thus does not perform pattern completion. Points above the main diagonal show that the output of the network is more similar to the stored pattern than the input was. So the network has performed some amount of pattern completion. The more the measurements are above the diagonal, the better the pattern completion performance (see Fig 3, right column). Measurements below the main diagonal indicate that the output of the network is on average less similar to the stored pattern than the input was, reflecting that information was lost during processing (see Fig 3 right column, RCN model).

To quantify the degree of pattern completion in a processing step, we define the pattern completion index (PCI) as the area between the main diagonal and the averaged output retrieval quality. Averaging was performed in 10 bins in input retrieval quality. The area is multiplied by a factor of 2 to obtain numerical values of the PCI between −1 and 1. Positive values imply that the network performs pattern completion, whereas negative values show that the network loses information. Values close to zero imply that the processing step does neither.

#### Sequence memory capacity

We further study the capacity of the CA3 network as well as the complete circuit, and estimate the number of patterns (sequences) the network is able to store and retrieve. For the specific number of stored sequences, we calculate the pattern completion index (PCI) for CA3-CA3 projections and end-to-end retrieval (EC_1_ − EC_M_), respectively. We define the network capacity in our model as the maximum number of sequences that can be stored in the network such that this PCI ≥ 0.

#### Robustness against dynamic noise

To make our neuron model more biologically plausible, we also add noise to the neural dynamics and investigate its effect on sequence retrieval. In these cases, Eq 5 is rewritten as

$$\begin{array}{c} h^{i}=\sum_{j=1}^{N_{\text{in}}}w^{ij}u^{j}+\varepsilon^{i}\left(0,\sigma^{2}\right), \end{array}$$ (12)

where *ε*^*i*^ is independent Gaussian noise with zero mean and variance *σ*^2^. The noise term is present in the dynamics both during storage and retrieval. We exclude this term for EC input neurons since we control the amount of noise added to the recall cue explicitly.

## Results

### Sequence completion in the CA3 network

#### Recall performance in CA3

We first investigate the ability of the network to retrieve the stored sequences when initialized with a noisy cue. Fig 3 (left column) shows the performance of the three different network models (DDN(*α*), RCN, and LCN) for a cue quality of *C*_cue_ = 0.4 which is initialised in EC input. In the subplots, each line indicates the retrieval quality for one sequence as a function of time. For *α* ≤ 0.9, i.e., if CA3 still has some recurrent dynamics during storage, the DDN retrieves the entire sequence almost perfectly. It is thus able to perform pattern completion and reach a retrieval quality of about 1, even when retrieval is initiated with a corrupted cue via EC, $C_{\text{CA}3}\left(y_{l,1},{\tilde{y}}_{l,1}\right)≃0.3$. However, the DDN is quite sensitive to noise for *α* > 0.9 and does not maintain the high retrieval quality for all sequences. At its maximum value *α* = 1, when the CA3 inherits the spatial correlations from the correlated grid inputs, memory performance of the network decreases abruptly. In this extreme case, the network model cannot even retrieve one of the stored sequences.

The reason for this difference in network performance for different values of *α* is not simply that intrinsically driven CA3 networks perform better. For instance, two other intrinsically driven CA3 networks do not perform well. The RCN is overall highly sensitive to noise as the retrieval quality is generally low (Fig 3, top-left). By contrast, the LCN, which allows controlling the correlation between successively stored patterns and is able to generate a continuously moving bump of activity, performs moderate pattern completion and maintains the retrieval quality for the remainder of the sequence (Fig 3, bottom-left). In this example, the adaptation parameter is *J* = 0.33. The slower the bump moves, the more robust the network is.

The results for these networks are summarized for the range of noise-levels, *C*_cue_ ∈ {0, 0.2, 0.4, 0.6, 0.8, 1}, in the PCI plots (Fig 3, right column). The DDN(*α* ≤ 0.9), performs sequence completion as the data points are well above the diagonal (PCI > 0). To examine the influence of plasticity in the recurrent CA3 synapses on the generation of robust sequences in CA3, we switch off plasticity in CA3 in the DDN(0) model. This model is equivalent to the randomly connected network (RCN). For the RCN, the data points lie below the diagonal, i.e., the network does not perform sequence completion (PCI = −0.1). Since sequence completion is crucial for the complete loop performance, we exclude this model from the following analyses. For the LCN, with low moving bump speed (*J* = 0.33), the data-points lie mostly on the diagonal (PCI = 0.03), suggesting that the network model largely maintains the information from the input cue. To conclude, the performance of recurrent networks in generating robust spatio-temporal sequences highly depends on their structure.

#### Impact of correlation on CA3 sequence dynamics

**Fig 4:**
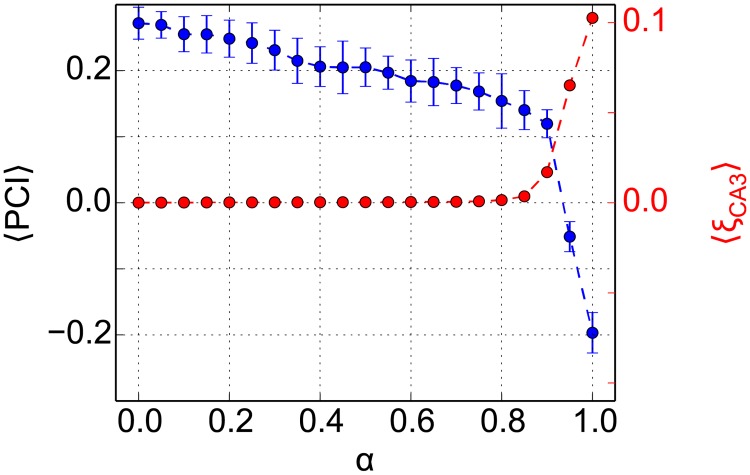
Correlation between stored patterns affects sequence completion in the DDN model. The average PCI value, which is calculated based on the results in Fig 3, right column, are shown in blue. The average proportion of large spatial correlations > *C*^*th*^ between any pair of stored patterns in CA3, 〈*ξ*_CA3_〉, are shown in red. Errorbars indicate the standard deviation across 20 repetitions of the simulation. Note the strong anti-correlation between 〈*ξ*_CA3_〉 and 〈PCI〉, particularly, the sharp increase and decrease, respectively, around *α* ≃ 0.9.

Since the external drive from grid-cell inputs on CA3 introduces correlations into CA3, we hypothesize that the DDN performs poorly for *α* ≥ 0.9 because the CA3 patterns are correlated. We therefore examined the spatial correlations between pairs of stored CA3 patterns, and quantified the proportion of large correlations, i.e., higher than *C*^th^ = 0.1.

$$\begin{array}{c} \xi_{\text{CA}3}=\frac{\sum_{\left\{l,m\right\}\neq \left\{l^{\prime },m^{\prime }\right\}}\Theta \left(C_{\text{CA}3}\left(y_{l,m},y_{l^{\prime },m^{\prime }}\right)-C^{\text{th}}\right)}{P(P-1)}, \end{array}$$ (13)

where Θ() is the Heaviside function and *P* = *L* × *M* is the total number of stored patterns in CA3. This proportion depends on curvature and intersection of trajectories that the simulated animal traverses in the environment. The average *ξ*_CA3_ is at or near zero for *α* ≤ 0.9 and sharply increases for larger values of *α* (Fig 4). To put these spatial correlations into perspective, we compared CA3 to EC and CA1. As expected, the proportion of large correlations is higher in EC, 〈*ξ*_EC_〉 ≃ 0.3, than in CA3, since EC patterns are generated by periodic grid cells. In CA1, the proportion of large correlations, 〈*ξ*_CA1_〉 ≃ 0.12, is comparable to CA3 in DDN(1), but lower than in EC. So, the random EC-CA1 projections decorrelate the patterns, which would be the same for CA3 in the DDN(*α* = 1) model. The powerful decorrelation effect of random projections also accounts for the near zero average *ξ*_CA3_ in CA3 for the DDN(*α* ≤ 0.9). Adding a small contribution from the initially random recurrent CA3 connections is sufficient to completely decorrelate the EC inputs in CA3.

Furthermore, the dependence of the average *ξ*_CA3_ on *α* is mirrored by the average PCI value, which sharply decreases around *α* ≃ 0.9. The anti-correlation between the average *ξ*_CA3_ and the average PCI suggests that it is the spatial correlation between patterns in the sequence that interferes with sequence retrieval. This result makes sense because the subsequent patterns in CA3 are hetero-associated according to Eq 11 and hetero-associations are known to be sensitive to correlations between the patterns.

Including the LCN model helps us better understanding the effect of correlation on the pattern completion. With *J* = .33, the average *ξ*_CA3_ = 0.16, is higher than for the DDN because of the continuous movement of the bump across the network. However, since the LCN does not engage synaptic plasticity, it does not suffer from the same interference problem as the DDN for *α* ≥ 0.9. The LCN neither performs sequence completion, nor does it lose information, as is evident from its low PCI of 0.03 and Fig 3. The low sequence memory performance of the LCN is the result of the continuous attractor network dynamics. Any activity bump is marginally stable—until the adaptation term in the LCN forces the bump to move. This property allows the network to remove noise deviations from the bump attractor, but it also makes it impossible for the CA3 network to correct an incorrect bump location once it has formed.

In summary, we find that the robustness of sequence generation depends sensitively on the dynamics of CA3 and that the reasons for low performance on sequence completion can be quite different for different networks. To achieve robust sequence completion in CA3 with correlated EC inputs, the sequential patterns in CA3 must be decorrelated.

#### Sequence memory capacity and the effect of dynamic noise on CA3 network

**Fig 5:**
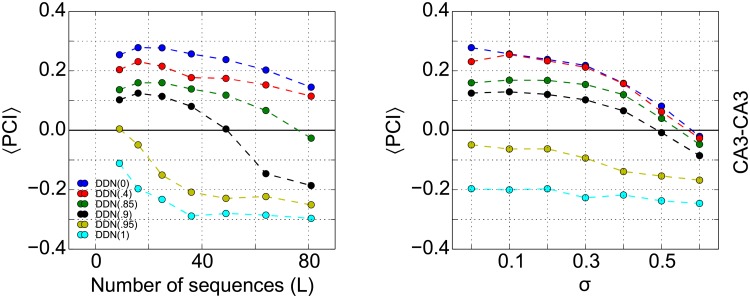
Systematic analysis of sequence memory capacity and the effect of noise on DDN. **Left**: Recall performance as quantified by the pattern completion index (PCI) in CA3-CA3 projections, for the six different DDN networks as a function of the number of stored sequences. We define the CA3 capacity in our model as the maximum number of sequences that can be stored such that PCI > 0. **Right**: DDN networks can tolerate retrieval noise injected into the neuronal dynamics (see Eq 12). PCI values are averaged across 20 repetition of the simulation.

To assess the biological plausibility of the DDN as a model for CA3, we study how many sequences the CA3 network in our particular model can store and whether the neural dynamics is robust to noise in the neural dynamics. The general sequence memory capacity of recurrent networks has been studied previously in idealized models in much greater detail (e.g., [63, 64]). We determine the CA3 capacity by calculating the average PCI for DDN networks, while increasing the number of stored sequences (Fig 5, left). The colors depict the results for an CA3 DDN(*α*) model for different values of *α*. There is no evidence for an abrupt change in retrieval quality as more and more patterns are stored, which would be evidence for catastrophic interference. Instead, retrieval quality degrades gracefully. The DDN(*α* > .9) does not reach our criterion (PCI > 0) at all and thus has zero capacity. The DDN(*α* < .9) has a capacity of at least around 70 sequences (about 1000 patterns), which is compatible with previous studies [12, 65].

When noise is added to the network dynamics in CA3, the network performance degrades gracefully (Fig 5, right). The recall performance remains remarkably constant up to *σ* ≃ 0.3, suggesting that the dynamics of the network is robust against low to moderate noise. In this simulation, we stored 256 patterns (16 sequences) in the network.

**Fig 6:**
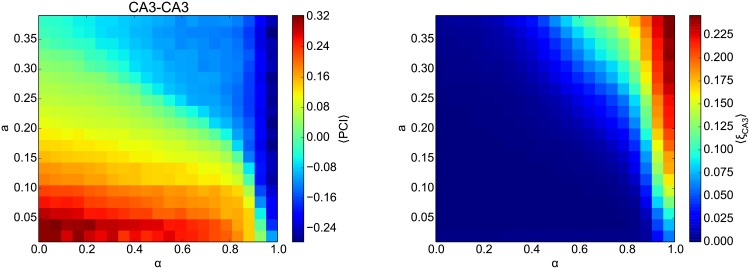
Systematic analysis of the effect of the pattern sparsity on sequence memory storage. **Left**: The average PCI (indicated by the color scale) in CA3-CA3 projections for different DDN networks as a function of the mixing parameter *α* and the sparseness of the patterns *a* (see Fig 1). Sixteen sequences were stored. Based on our criteria, the CA3 network is able to successfully store and retrieve memory sequences when PCI > 0. PCI values are averaged across 20 repetition of the simulation. **Right**: The average proportion of large correlations between pairs of stored patterns, 〈*ξ*_CA3_〉. The patterns in both panels confirm that PCI memory retrieval is only possible, i.e., PCI > 0, if 〈*ξ*_CA3_〉 is near zero.

Treves and Rolls (1992) [65] reported that the recurrent network capacity is proportional to the number of modifiable synapses per cell, with a factor that increases roughly with the inverse of the pattern sparsity *a*. However, when we performed simulations with all-to-all or 10% connectivity (data not shown), we found no qualitative difference in the results, indicating that our results are not sensitive to the number of synapses within the range tested here. The most important parameter that can significantly affect the network capacity is the sparsity of the stored patterns (Fig 6, left). To confirm the role of correlations between patterns in CA3 on memory performance, we calculated *ξ*_CA3_ (Fig 6, right). The patterns in both panels confirm that PCI memory retrieval is only possible, i.e., PCI > 0, if 〈*ξ*_CA3_〉 is near zero. Another way to interpret the results in Fig 6 is the following: The denser the CA3 patterns are, i.e., the higher *a*, the lower the mixing parameter *α* has to be for the memory network to perform. Put differently, the DDN networks require more CA3 intrinsic contribution (lower *α*) to decorrelate the inputs patterns when the patterns are denser (larger *a*).

### Storing and retrieving sequences in the hippocampal circuit

**Fig 7:**
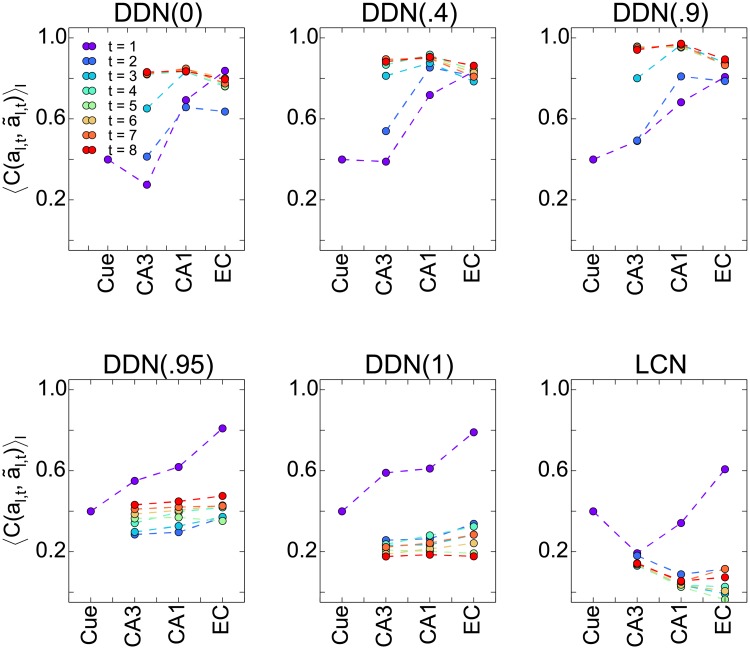
Average retrieval performance at different processing stages in the hippocampal circuit. Each panel shows the average correlation between retrieved and originally stored patterns for a different CA3 model. The data indicate that pattern completion occurs in the EC-CA3, CA3-CA3 (through time), CA3-CA1 and CA1-EC projections. Each data point, except for the retrieval cue, shows the average performance in the network layer. Different colors indicate the performance for the eight first time steps. In all panels, the cue quality was *C*_cue_ = 0.4. Note that our network consists of deep and superficial layers of the EC, which are clamped to the same activity during learning, but not during retrieval. During retrieval, we only provide the input cue to the superficial layer and it is completed through the EC(superficial)-CA3-CA1-EC(deep) pathway. The “cue” is applied to the superficial layers of EC and “EC” in the panels refers to output of the network in the deep layers of EC.

We found previously that pattern completion in feedforward networks is important in a model of the hippocampal formation and that the statistics of CA3 patterns are important for memory performance [43]. Since our current study suggests that the DDN(*α* ≤ 0.9) models work best for sequence completion in CA3, we studied how the generated CA3 pattern statistics affect pattern completion in the feedforward network. To this end, we retrieved the sequence based on a noisy retrieval cue of the first pattern (see Methods) and then averaged over the retrieval quality in each time step, ${\langle C\left(a_{l,t},{\tilde{a}}_{l,t}\right)\rangle }_{l}$, in different processing stages of the loop, namely EC (cue), CA3, CA1, and EC (output) (Fig 7). Five panels each illustrates the results for a DDN(*α*) model with different values of *α*. The most obvious contrast is the comparison between DDN(0) and DDN(1). For intermediate values of *α*, the DNN(*α*) network shows a behavior similar to either DDN(0) or DDN(1), with an abrupt change around the familiar transition point of *α* ≃ 0.9. In the first time step, DDN(0) loses information in the hetero-association from EC to CA3, but the next two feedforward stages perform pattern completion through the CA3-CA1 and CA1-EC projections and more than compensate for the initial loss of information. In later time steps, the retrieval quality in CA3 steadily increases due to sequence completion in CA3. Both sequence completion and pattern completion in the feedforward circuit eventually saturate as retrieval proceeds in time and through the processing stages, respectively. The results for DDN(1) are quite different. In the first time step, it consistently performs pattern completion through all processing stages, including from EC to CA3. Since this latter point markedly differs from DDN(0), and will be studied in more detail below. Due to the inability of the CA3 recurrent dynamics in DDN(1) to retrieve the second, and later, elements in the sequence, retrieval performance in CA3 drops dramatically relative to the first time step.

For the LCN model, a different pattern of retrieval quality across different processing stages and time emerges (Fig 7, bottom-right panel). Pattern completion of the first pattern looks roughly similar to DDN(0), but pattern completion of later patterns looks more like that of DDN(1). Note that in CA3, the retrieval quality for *t* ≥ 2 does not significantly differ from that for *t* = 1, but the subsequent feedforward pattern completion differs greatly. This suggests that retrieval quality, the correlation between retrieved and stored patterns, in CA3 is not sufficient to characterize the retrieved pattern completely. In other words, two different noisy patterns that have the same retrieval quality can nonetheless subsequently yield different feedforward pattern completion. One type of noise slightly degrades pattern completion, the other type destroys it. Our analysis in section suggest that random fluctuations of the bump degrade pattern completion, whereas shifts of the bump are destructive. With the LCN model, CA3 can generate bump patterns that might never have been stored in the network. Therefore the downstream CA3-CA1 projection cannot decode the information. By contrast, the DDN(*α* ≤ .9) models generate uncorrelated patterns in CA3 and store them, which allows for better retrieval and subsequent pattern completion in the feedforward network.

**Fig 8:**
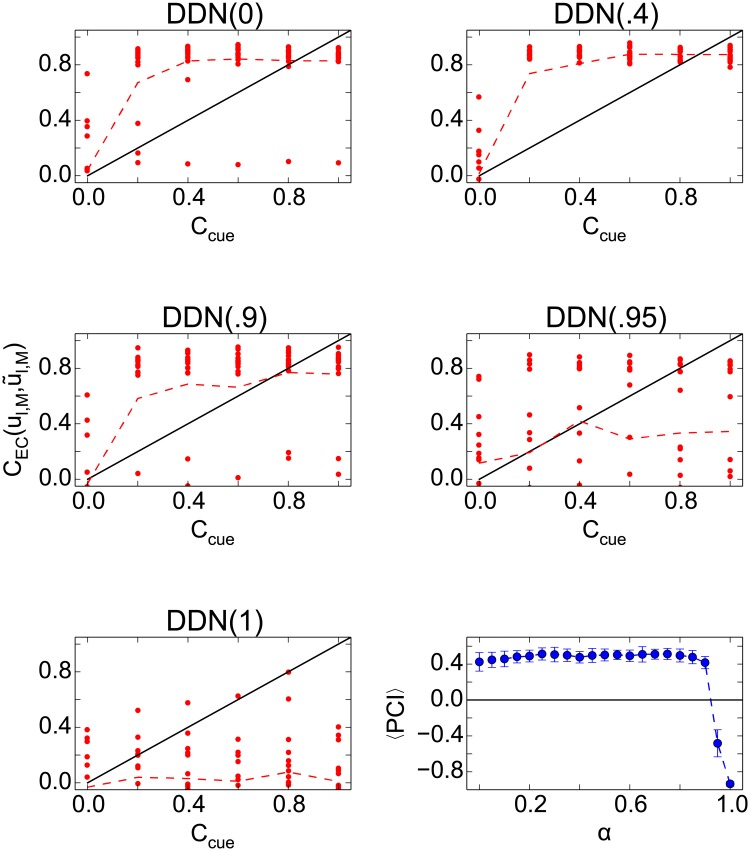
End-to-end retrieval performance. The retrieval correlations of the last sequence element in the output is compared to the retrieval quality of the cue in the input. Each panel corresponds to a DDN model with different *α*. The panel in the bottom-right illustrates the average PCI value against the parameter *α*.

To assess the overall retrieval quality systematically, we analyzed the end-to-end retrieval performance by comparing the retrieval correlations of the last sequence element in the output $C_{\text{EC}}\left(u_{l,M},{\tilde{u}}_{l,M}\right)$ to the retrieval quality of the cue in the input (*C*_cue_) (Fig 8). The DDN(*α* > .9) models of CA3 fail to retrieve the stored sequences at any recall cue quality. Whereas with the DDN(*α* ≤ .9) in CA3, the model performs excellent sequence completion for the recall cue qualities > 0.2.

#### Sequence memory capacity and the effect of dynamic noise on pattern completion

**Fig 9:**
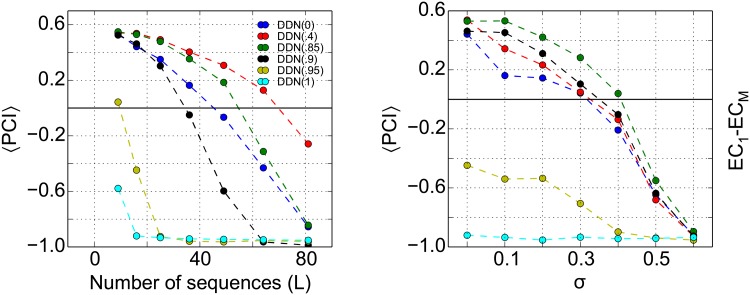
Memory capacity of the hippocampal circuit measured by the end-to-end memory performance. **Left**: Shown is the average PCI when comparing the retrieval quality of the first and last patterns of the sequences in EC, for six different DDN networks as a function of the number of stored sequences. The capacity degrades gracefully. **Right**: Effect of noise in neural dynamics on overall network performance when *L* = 16 sequences are stored.

For a memory circuit to be useful in practice, its overall performance should have sufficient capacity and degrade gracefully when the capacity is exceeded or noise is present in the network dynamics. We, therefore, study the capacity of the network based on the end-to-end retrieval performance (Fig 8). For the specific number of stored sequences, we calculated the average PCI and define the network capacity in our model as the maximum number of sequences that can be stored in the network such that this PCI > 0. Indeed, the retrieval quality degrades gracefully in all DDN(*α* ≤ 0.9) models with increasing memory load and noise (Fig 9). The networks have a capacity of ≥ 25 sequences, i.e., ≥ 400 patterns. As expected from the data presented so far, DDN(1) has zero capacity. The capacity limit for end-to-end retrieval follows an inverted U-shape, initially increasing with *α* and then decreasing again. Similarly, the robustness to noise does not follow a simple rule. Our data indicate DDN(0.85) is the most robust. These results contrast with those for sequence completion in CA3, where the capacity limit decreases monotonically with *α* and DDN(0) is the most robust to noise (Fig 5).

**Fig 10:**
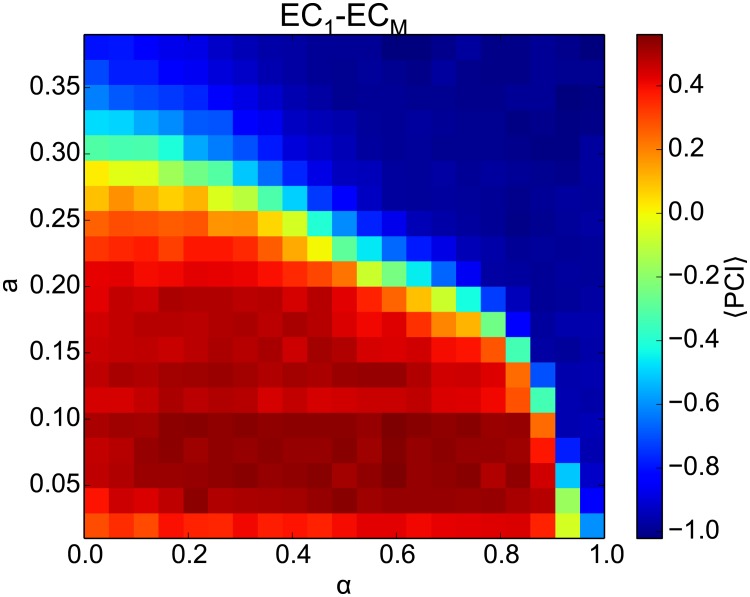
The effect of the pattern sparsity on the end-to-end memory performance. Average recall performance 〈*PCI*〉 in the complete loop for different DDN networks as a function of the pattern sparsity *a* and *α*. Note that the *a* parameter is changed only for the CA3 network.

We also investigate the overall network performance with respect to the patters sparsity *a* (Fig 10). For the fixed number of stored sequences *L* = 16, we calculated the average PCI for different DDN(*α*) models, while increasing the parameter *a*. Comparing the results to that of Fig 6, reveals the effect of spatial correlation between successive patterns on the overall network performance.

Even though we found that pattern completion through hetero-association in feedforward networks is sensitive to the CA3 dynamics, it is nevertheless functional for the DDN model of CA3. The results, therefore, show that the sequential retrieval in CA3 can be combined with feedforward pattern completion, which is one of the core assumptions of CRISP.

### The effect of the pattern statistics on pattern completion

Our results reveal that correlations between CA3 pattern have a complex influence on feedforward pattern completion in the hippocampal circuit. In particular, it is intriguing that not all processing steps perform well in all cases (see Fig 7). To better understand the causes behind the different performance in pattern completion, we investigated the manifolds in which the stored patterns in EC, CA3, and CA1 lie. The input dimensionality of the patterns in each layer equals the number of cells *N*. The subspace, in that the *P* (= 256) stored patterns lie, has a dimensionality of at most *P*. However, due to the correlation between the stored patterns, the number of significant dimensions can be much lower. For instance, since the manifold of EC patterns are generated from grid cells as the simulated animal moves in a 2-d space, the EC patterns form a 2-d manifold, albeit a highly nonlinear one. Here, we use principal component analysis (PCA) to get an impression of what the pattern manifolds in the different layers of our network look like. PCA identifies orthogonal directions (principal components) in the pattern space such that the variance of the data along these principal components is maximal. This way, PCA redistributes the data such that the first *k* components explain as much of the total variance as possible.

**Fig 11:**
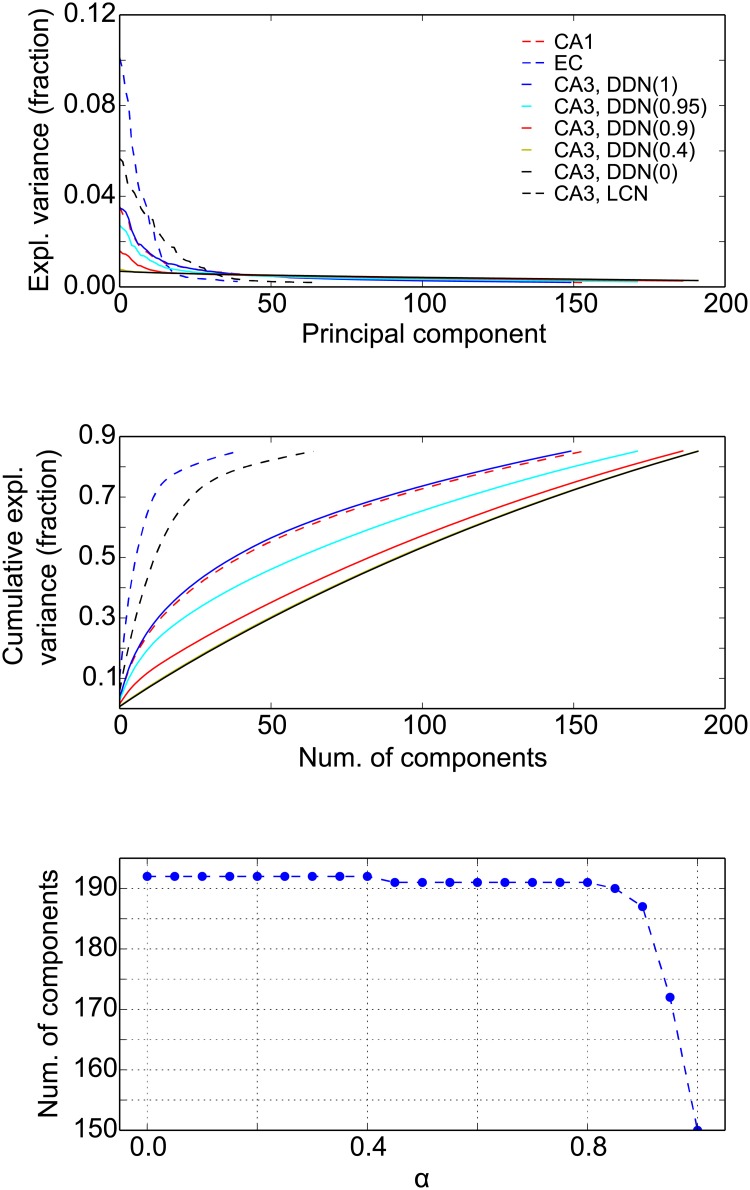
Dimensionality of the pattern manifold in different layers. The variance of the stored patterns explained by principle components (PCA) in EC, CA1, and CA3 for different network models. **Top**: Fraction of the variance explained by individual components. **Middle**: Cumulative fraction of explained variance. **Bottom**: Number of components that are required to explain at least 85% of the variance in CA3 for DDN models as a function of parameter *α*. Note how this number suddenly drops around *α* = 0.9.

Analysis on the grid patterns in the EC layer of our model indicates that only few components explain any variance (Fig 11, top), cumulatively, 40 dimensions explain about 85% of the total variance (Fig 11, middle). The estimated dimensionality is higher than the theoretical value of 2, but since PCA is a linear method, we cannot expect it to be able to identify the highly nonlinear 2-d manifold. For our purpose, it suffices that PCA shows that EC patterns lie on a relatively low dimensional manifold. PCA also reveals that CA3 patterns in the LCN are low dimensional, consistent with the fact that network activity is constrained by the attractor dynamics to a 2-d manifold. Patterns in CA1 and in CA3 in the DDN(1) model also lie in a lower dimensional space (Fig 11), since they are directly driven by EC. Nevertheless due to the decorrelating effect of the EC afferent projections, the dimensionality is higher than in EC. The DDN(0) patterns have the highest dimensionality, since the patterns are generated purely by the random recurrent connections in CA3 and are nearly orthogonal to each other. As *α* increases from zero, the number of components that are required to explain at least 85% of the variance in the DDN(*α*) initially remains constant, but suddenly drops around *α* ≃ 0.9 (Fig 11, bottom). These results are consistent with our findings on the *ξ*_CA3_ (Fig 4), indicating that the spatial correlations between the neural patterns arise from a movement along a trajectory in a low dimensional manifold.

**Fig 12:**
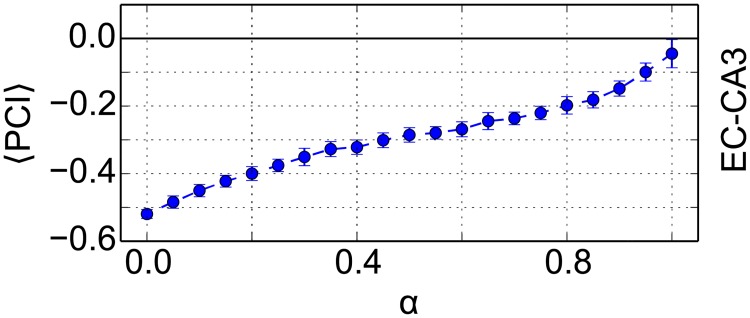
Pattern completion in EC-CA3 projections. Average pattern completion index (〈*PCI*〉) for EC-CA3 increases with the mixing parameter *α*.

The analysis of the dimensionality also raises an apparent paradox. If the dimensionality of the pattern space in CA3 decreases with *α* (Fig 4), the spatial correlations increase accordingly. Higher correlations should deteriorate hetero-association in the EC-CA3 projections leading to a decreased memory performance with increasing *α*. However, the opposite is the case. This is already visible in Fig 7, where pattern completion from EC to CA3 improves with increasing *α*. The average PCI for the EC-CA3 projections shows more clearly that the PCI indeed increases with *α* (Fig 12), yet overall memory performance decreases with *α* (Fig 8).

We hypothesize that the apparent paradox arises because the correlations between retrieved and stored patterns, the retrieval quality, alone are insufficient to fully characterize memory performance of a network. We discussed similar cases in our previous work [43] and above, where very similar retrieval qualities in CA3 nevertheless sometimes lead to pattern completions and sometimes to information loss (Fig 7, bottom-right panel, discussed in section). What matters for hetero-association is that one particular pattern in the sending layer is uniquely associated with one pattern in the receiving layer. If input and target patterns are random and uncorrelated, we have pure associative memory [44]. When input or target patterns are strongly correlated, i.e., patterns are less distinct from one another, it becomes more difficult to uniquely hetero-associated input and target patterns. However, in this case the retrieval quality might still be high. Consider the extreme case where all the target patterns were identical, then retrieval quality would be perfect (1) for every retrieval, but the target pattern contains no information whatsoever about the input pattern and therefore cannot serve for memory retrieval downstream.

**Fig 13:**
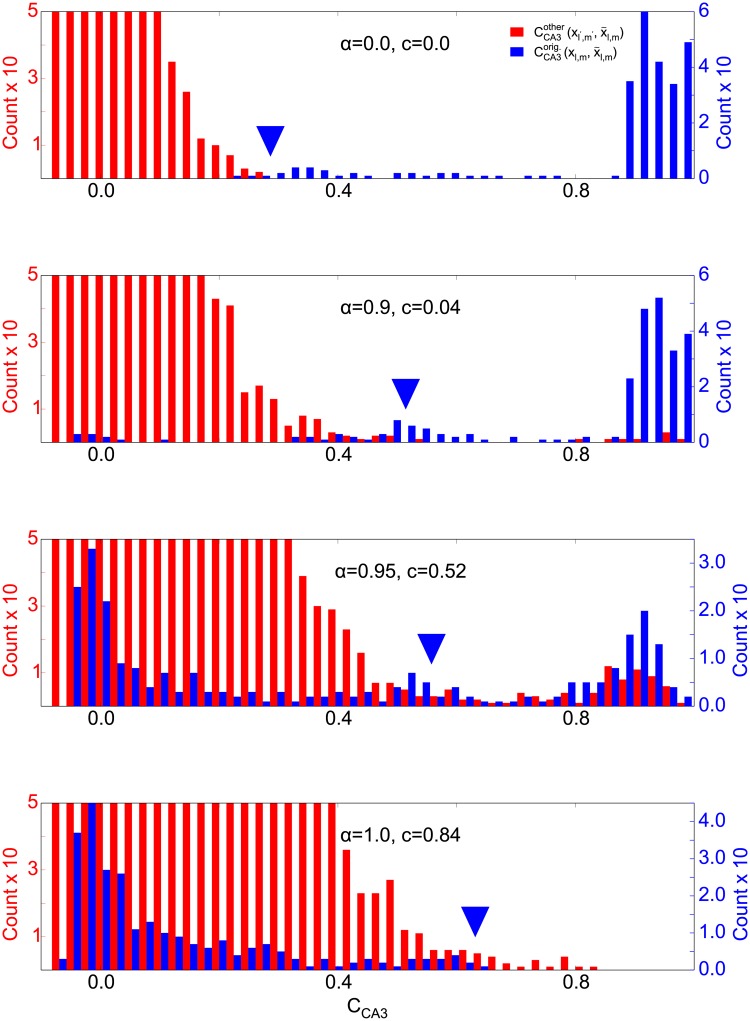
How distinct are correct CA3 patterns from incorrect ones? Histograms of correlations between retrieved patterns and corresponding stored patterns (blue) and between retrieved patterns and all other stored patterns (red). Retrieval is initiated with the perfect recall cues in EC. The number *c* inside each panel shows the confusion rate. That is how often the correlation between the retrieved and the original pattern is smaller than the correlation between retrieved pattern and at least one of the other stored patterns in the network. The large triangle marks indicate the average cue quality ${\langle C_{\text{CA}3}\left(y_{l,1},{\tilde{y}}_{l,1}\right)\rangle }_{l}$ that initializes CA3 dynamics during the retrieval process. For *α* ≤ 0.9, there is little to no overlap between the correct CA3 patterns and incorrect ones.

The correlations between CA3 patterns in our DDN(*α* ≥ 0.9) are less severe than in this extreme example, but the extreme example is nevertheless a useful guidance to understanding pattern completion in the feedforward circuit. So we hypothesize that, for DDN(*α* ≥ 0.9), retrieved patterns in CA3 are less informative about the input, even though their correlation to the correct pattern is higher. We therefore compared the correlations between retrieved CA3 patterns and the corresponding original pattern, $C_{\text{CA}3}^{\text{orig}}\left(y_{l,m},{\tilde{y}}_{l,m}\right)$, to the retrieved patterns and the other stored patterns, $C_{\text{CA}3}^{\text{others}}\left(y_{l^{\prime },m^{\prime }},{\tilde{y}}_{l,m}\right)$ where {*l*′, *m*′} ≠ {*l*, *m*}). Hetero-association is successful, if the two distributions are distinct from one another. This is indeed the case for *α* ≤ 0.9, but not for *α* ≥ 0.9 (Fig 13), confirming our hypothesis. The retrieval in these simulations was initiated with the perfect recall cue in EC. Interestingly, for DDN(*α* ≤ 0.9) the average retrieval quality of the pattern that initializes retrieval in CA3, ${\langle C_{\text{CA}3}\left(y_{l,1},{\tilde{y}}_{l,1}\right)\rangle }_{l}$ (triangles in Fig 13) almost have zero overlap with the $C_{\text{CA}3}^{\text{others}}$ distribution. This allows the CA3-CA1 projection to perform hetero-association with the CA1 pattern and CA3 to perform sequence completion to retrieve the next elements of the stored sequence, even though the average retrieval quality is rather low. On the other hand, for DDN(*α* ≥ 0.9) the retrieval quality is much higher, but the initially retrieved pattern overlaps with the $C_{\text{CA}3}^{\text{others}}$ distribution, making the cue less informative. The overlap can be quantified by the confusion rate, i.e., how often the correlation between the retrieved and the original pattern is smaller than the correlation between the retrieved pattern and at least one of the other stored patterns in the network (variable c in Fig 13). Because of the high confusion rate for *α* ≥ 0.9, the CA3-CA1 projections in these networks cannot decode the stored patterns in CA3 and therefore patterns completion fails. Furthermore, the recurrent CA3 dynamics fails to retrieve the subsequent elements of the stored sequence in these cases.

To summarize, the pattern statistics imposed by the network structure and dynamics in CA3 has an important and complex influence on the hippocampal circuit’s ability to perform pattern completion. Correlations between stored patterns deteriorate CA3 sequence retrieval as well as feedforward hetero-association. However, the frequently used correlation between retrieved and original pattern is insufficient to characterize memory performance of the network, or of its subparts, because this correlation does not indicate how informative the retrieved pattern is about the input pattern.

## Discussion

We have investigated the storage and retrieval of memory sequences in the hippocampal circuit based on the recently proposed CRISP theory. CRISP is built around intrinsic sequences in CA3 and pattern completion in feedforward projections. We confirmed many assumptions of CRISP, which were previously argued for based on experimental results and intuition, in computational models of the hippocampal circuit. We found that the network model can store and robustly retrieve sequences of realistic EC inputs. Memory performance critically depends on the network dynamics in CA3, as already proposed by CRISP. In a prior study, we have shown that, for realistic inputs pattern, completion through the feedforward network is a superior alternative to auto-association in CA3. Here, we confirmed that the hippocampal circuit can perform pattern completion with sequential CA3 dynamics in the loop. Furthermore, correlations between stored patterns in CA3 deteriorate both sequence completion in CA3 and pattern completion in the feedforward projections. We also found that, having good retrieval performance in CA3 does not necessarily mean that retrieval performance of the complete loop will be good as well. In some cases, a lower performance in CA3 can yield better overall memory performance.

### Network structure and plasticity in CA3

It turns out that the best performance in our study was achieved by the DDN model for CA3 with *α* ≤ 0.9. In contrast to CRISP, which postulated no plasticity in CA3 during learning, this model requires plasticity in CA3 during the learning phase. How can this discrepancy be explained? There are several options. First, to salvage the original postulate, one could assume the DDN(0) model for CA3 and assume that plasticity in CA3 occurs in a separate pre-training phase. Since the activity in the DDN(0) model is independent of the input, the activity could be triggered in the absence of any input. Then plasticity in the recurrent CA3 connections would not be needed during the later learning phase. Second, it might be possible that a static CA3 model, other than the RCN and LCN studied here, could provide even higher memory performance than our DDN(*α* ≤ 0.9) models, and we simply have not found this model, yet. Third, we could drop the assumption of no plasticity in CA3 during learning from CRISP. This assumption was based on experimental reports (e.g., [66]), but since their initial publication, there have not been many follow-up studies. So, the strength of the initial results remains untested.

We would like to emphasize that despite the uncertainty about plasticity in CA3, our results suggest that CA3 patterns should be decorrelated and somewhat independent of the EC input. The latter confirms another assumption of CRISP that CA3 sequences are intrinsically generated and not imprinted by external inputs. Intrinsically generated sequences have been observed in a number of different studies. During the delay period in an ongoing task, hippocampal neurons fire in a reproducible temporal sequence [67, 68]. Sequential activities were observed in an offline state before rodents explore a novel environment, which were correlated with the ordering of place fields in the novel environment (preplay) [69]. This preplay phenomenon suggests that the offline sequences could not have been established by external sensory inputs and are intrinsic to CA3 [46].

Based on our results, it is difficult to predict which one of the tested network architectures is more likely to resemble the hippocampus anatomy. We have tried to incorporate the anatomical parameters that are most often measured, i.e., neuron numbers, neural activity levels and degree of connectivity, in all models. Only recently, higher-order anatomical features have been studied (e.g., [15]). While we believe that it is too early to exclude a model merely based on their results, they do suggest that a random connectivity is unlikely.

### The function of DG

In our network, we did not include the DG explicitly, but the influence of DG can be integrated into our model. A number of studies have indicated that DG orthogonalizes the patterns before storage in a process known as pattern separation [10, 11, 13, 70]. Pattern separation is facilitated by sparse activity in DG, sparse DG-CA3 connectivity and adult neurogenesis in DG. The latter effect results because newborn granule cells have little overlap with older DG cells with respect to their projections to CA3 [71–73]. According to CRISP, this pattern separation and strong drive from DG onto CA3 are particularly important for initiating the CA3 sequence during memory storage and retrieval. Sequence initiation is not implemented using neural mechanisms in this study, but *ad hoc* using some form of random initialization, because we wanted to focus on sequence and pattern completion. DG could be responsible for this function, which will have to be studied in the future.

In our current model, uncorrelated CA3 patterns arise solely due to initial random weights, which partially drive the CA3 recurrent dynamics: the smaller *α*, the larger the influence. In a long-lived animal, this mechanism might not suffice and might have to be supported or supplanted by another mechanism. The DG might play a role in producing sequences of uncorrelated patterns in CA3. It is conceivable that a pool of uncorrelated sequences is established in the CA3 network when newborn neurons integrate into the DG network and provide orthogonal activity to CA3. In this scenario, adult neurogenesis would effectively affect the *α* parameter in our DDN model. The lower the rate of adult neurogenesis, the more correlated CA3 patterns would be, and therefore the higher *α*. This leads to a prediction for memory performance across the lifespan, since the rate of adult neurogenesis changes throughout the animal’s lifetime [74, 75]. Mice in middle age have about 80% fewer neural progenitor cell proliferation, neuronal differentiation, and newborn neuron survival than mice in early adulthood [76]. In the mouse DG, only 8.5% of the neurons born postnatally are added after middle age [77]. If our hypothesis is correct, then the *α* in middle and older age is larger than that during early adulthood. Since our model shows that memory performance is low for large values of *α*, our model predicts that the memory performance in middle and older age is inferior to that during early adulthood.

### Pattern completion in CA1

The standard framework does not offer a clear function for CA1, but some studies hypothesize that CA1 plays a role in novelty or mismatch detection [78, 79]by increasing its activity when rats are exposed to novel environments [80, 81]. This hypothesis does not however account easily for the general nature of memory deficits after lesions of CA1, e.g., in the retrieval of contextual fear conditioning [82], and spatial information [83–85]. By contrast, CRISP suggests that CA1 performs pattern completion of CA3 patterns to increase the precision and robustness of retrieval. This suggestion is well supported by our modelling results (e.g., see Fig 7). In a way, CA1 decodes the highly transformed patterns in CA3 back to their original versions in EC [43].

Alternative suggestions for the function of CA1 including the memory for temporal information [86], although the experimental evidence does not support a selective role of CA1 since they also report similar deficits in temporal pattern separation after CA3 lesions (e.g., see ref. 22 in [86]). In addition, there is experimental evidence that the loss of CA3 inputs abolished temporal coding (or sequence timing) in the CA1 population [87]. Furthermore, CA3 cells exhibit robust temporal modulation when animals performed a memory task [88], similar to the activity of time cells in CA1. We note that within the CRISP framework any function assigned to CA3 requires CA1, as well, since CA3 only projects to neocortex via CA1. Many other models agree with this notion. By contrast, Kesner hypothesizes that the direct projections from CA3 (and DG) to neocortex are more important than commonly appreciated and therefore each hippocampal subregion can be assigned its own independent function. Since this is an ongoing debate in the field, we unfortunately cannot resolve this issue at this moment. It is tempting to bring time cells to bear on sequence memory as we discuss it here, however, we feel that the time scale of the sequences would be too slow for memory retrieval.

### The temporal dimension

The dynamics of neural networks in the brain is continuous and any attractor network settles in the basin of attraction within 10-20 ms [89–91], although this time scale depends crucially on the time constants governing synaptic conductances. Nevertheless, this raises the question of how the hippocampus can associate events that are separated by seconds or more. As mentioned above, a potential solution could be that sequence of events on the behavioral time scale are represented by compressed neural sequences at the timescale of milliseconds [41, 92, 93] through theta phase precession [49, 50].

Another important issue is that our network requires a pacemaker to keep the retrieval process synchronized within and across the different subregions. This could be achieved by various oscillations (e.g. theta, gamma, ripples) that are present in the hippocampus and that have been linked to episodic memory and sequence learning in the hippocampus [94]. The most promising candidate for this pacemaker is probably the gamma oscillation (approx. 40-120 Hz), as suggested previously [40, 95–97]. The timescale of theta phase precession, which might serve to compress behavioral sequences on the timescale of seconds into the range of tens of milliseconds, fits well with the timescale of gamma oscillations. It is therefore possible that sequences of events are compressed by phase precession into timeslots of synchronized activity organized by gamma oscillations.

Finally, we emphasize that some aspects of our study were determined for convenience and clarity, not because the network depends on them. For instance, we always used the first patterns in the sequences as retrieval cues because that way the sequence of retrieved items is the longest. We could have initiated retrieval with any later element and the network dynamics would have been the same, except that the retrieved sequence would have had fewer items to analyze. While this would not have been an issue for many networks, for the last three networks in Fig 3, it could have made it difficult to discern the dynamics of the retrieval. Another aspect is the number of items from the sequence that are provided as retrieval cues. In our study, only a single input pattern was presented to the network to study the retrieval of the memory sequence through the hippocampal circuit, heavily based on sequence retrieval in CA3. If more input patterns representing later sequence elements were provided in EC, these would interfere with the subsequent patterns retrieved from CA3. This interference highly depends on the nature of the patterns that are used in EC, the performance of the EC-CA3 projections in pattern completion, and CA3 dynamics. Even though the network dynamics would be the same, the retrieved sequences in the output would be much harder to interpret since they would be mixtures of multiple processes.

### Relationship to spatial memory

In this study, we focused on the function of the hippocampal formation in sequence memory. However, spatial memory is also likely to be an ethologically relevant function of the hippocampus. For instance, the hippocampus is necessary for spatial learning in rodents [5] and humans [98]. Several types of cells in the hippocampal formation appear to encode spatial information, including head-direction cells [99], border cells [100], irregular spatial cells [101], place cells and grid cells. However, other cell types in the hippocampal formation encode nonspatial information, such as odor-sensitive cells [102], nonspatial cells [101], and time cells [68, 88]. This diversity of cell types is consistent with the function of the human hippocampus in episodic memory [98]. While the focus of this article was on episodic memory, our network stored spatial information from grid cells. The full range of inputs to the hippocampus and the mixture of different inputs are poorly explored. We expect that our results are applicable beyond grid patterns because it is the correlation between CA3 patterns that are detrimental to memory performance and similar correlations are present in any of the aforementioned cell types and quite likely in episodic memory patterns in general.

Our model could help to study whether the spatial representation in CA1 and CA3 can be reconciled with episodic memory in the same neural network model. We found previously that a fairly generic and robust solution to the transformation from grid cells to place cells could be learned in a feedforward model [103–105]. We also found evidence for spatial coding in CA1 and CA3 in a model related to the current one [43]. Since our model includes the hippocampal circuit, it enables future investigation of spatial representations in the hippocampal subregions.

### Relation to other studies

It is interesting to consider not only sequence storage and retrieval but also other types of sequence processing, such as intersecting sequences, the possibility of skipping some patterns, or even taking longer shortcuts, etc. As we illustrate in Fig 2A, some stored trajectories do intersect each other. Since CA3 adds a random pattern to the EC input pattern at each time-step, two identical or similar EC patterns are separated enough (when *α* ≤ 0.9) so that the network can disambiguate the overlapping patterns and retrieve the right sequence. However, jump-ahead or shortcut recall would require reward or some other mechanism to define the goal (as, for example, in [38]), which is not included in our model.

Previous studies have shown that hippocampal sequences can be produced by highly structured neural circuits, e.g., continuous attractor networks. Here a localized bump of activity in the neural tissue moves around because of asymmetric patterns of connectivity, short-term plasticity, or slow, local negative feedback [46, 106–108]. These highly structured circuits are less likely to produce models with flexible circuitry or to generate dynamics with the temporal complexity needed to account for experimental data [109]. In contrast, random networks have been modified by training to perform a variety of tasks [34, 110–113]. Liquid State Machines (LSMs) learn sequences in a completely different way than conventional recurrent neural network (RNN) systems [112]. LSMs use a dynamic reservoir to recode time-series data. After a certain time-period, the state of the liquid is used as an input for a readout network. This readout network learns to map the states of the liquid to the target outputs. This means there is no need to train the weights of the RNN, which decreases the computation time and, more importantly, the complexity of learning time-series data. The LSM model provides a framework to analyse continuous streams of input. Given a time series as an input, the LSM can produce a time series of behaviors as an output. The desired behavior can be achieved by adjusting the weights on the links between the reservoir and the output [114]. While these models can flexibly learn many different tasks, the assumption of chaotic spontaneous activity in a constant recurrent network is less biologically realistic.

Sussillo and Abbott developed a training scheme called FORCE learning that reorganizes the chaotic spontaneous activity of a recurrent network into coherent activity patterns required to generate controlled actions [34]. In this case, a recurrent generator network drives a linear readout unit through weights that are modified during the FORCE training. It differs from traditional training in neural networks, where normally network parameters (i.e., synaptic strengths) are modified gradually on the basis of initially large output errors until a desired response is produced. In FORCE learning the output errors are small from the beginning of the training process. Therefore, the goal of training is not significant error reduction, but rather reducing the amount of modification needed to maintain these small output errors. After training, modification is no longer needed, and the network can generate the desired output autonomously. The FORCE learning also allows the modification of recurrent generator network synapses. This advantage makes the network architecture more biologically plausible. The network receives no external feedback, i.e., the feedback is generated within the network. The FORCE learning procedure has been compared to Jaeger and Hass’ echo-state learning where the recurrent network synapses are fixed and the desired output is fed back without noise to the network during training [113]. Here, feeding erroneous output back into a network during training modulates its activity, so that learning fails to converge. On the other hand, removing all feedback errors can lead to stability problem since it prevents the network from sampling fluctuations during training. The FORCE learning procedure is used to control the feedback signal and allows fluctuations to be sampled and stabilized. Overall, results show that echo-state learning converges less often and with larger error than FORCE learning. Even though in these models the random recurrent generator plays an important role in sequence learning, our model assigns much more critical function to it, namely sequence completion. FORCE learning is aimed at a pattern of activity in a read-out node, which computes the output of the recurrent network. Our network learns the input sequences reliably in one shot, but in FORCE learning the output is fed back to the network several times forcing the network to generate the desired sequence. In addition, sequence generation in these models is not guarantee to be robust to noise.

There are a number of biologically motivated sequence memory models that are more detailed than our neuron model [115, 116]. These models show how spike-timing-dependent plasticity (STDP) can lead to a cell becoming responsive to a particular sequence of presynaptic spikes and to a specific time delay between the spikes [117, 118]. Hawkins and Ahmad proposed a mechanism, in which a neuron can predict its activation in hundreds of independent contexts [119]. They then present a network model based on neurons with these properties that learns neural sequences. It remains to be seen whether our results on sequence memory in the hippocampal circuit are reproducible using more biologically plausible model components, e.g., spiking neurons and STDP learning rule.

### Predictions

Our modeling results make the strong prediction that the spatial correlations between patterns in CA3 is lower than those in EC or CA1, which could be easily tested in existing experimental data. To our knowledge, the closest that a reported result has come to test this prediction is a study by Leutgeb et al [120]. They have found that the CA3 population activity had lower overlap between two different environments than the CA1 population activity, whereas between two exposures to the same environment the overlaps were the same in CA1 and CA3. However, their overlap measure was based on the ratio of the average firing rates in the two environments (or exposures) and therefore does not allow any insight into the individual patterns in CA3 and CA1. Furthermore, in our model CA3 is important for sequence learning, whereas CA1 performs pattern completion. This can be tested experimentally and compared with the predictions of the standard framework. One can train an animal on tasks that require either associative or sequence learning. Our model predicts that a CA3 lesion prevents the animal from learning the sequential task, but it does not affect associative learning. The standard framework predicts the opposite outcome.

### Conclusion

Compared to previous models, CRISP proposes a different mechanism for storing episodic memories in the hippocampus. Neural sequences are generated in CA3, and inputs are mapped onto these sequences through synaptic plasticity in the feedforward projections of the hippocampus. Here, we used computational models to confirm that CRISP is a viable theory for episodic memory storage in the hippocampus, but more work is required to test all aspects of the theory.
